# Ostarine-Induced Myogenic Differentiation in C2C12, L6, and Rat Muscles

**DOI:** 10.3390/ijms23084404

**Published:** 2022-04-15

**Authors:** Natalia Leciejewska, Paweł A. Kołodziejski, Maciej Sassek, Leszek Nogowski, Emilian Małek, Ewa Pruszyńska-Oszmałek

**Affiliations:** 1 Department of Animal Physiology, Biochemistry and Biostructure, Poznan University of Life Sciences, 60-637 Poznan, Poland; natalia.leciejewska@up.poznan.pl (N.L.); pawel.kolodziejski@up.poznan.pl (P.A.K.); maciej.sassek@up.poznan.pl (M.S.); leszek.nogowski@up.poznan.pl (L.N.); 2 Department of Preclinical Sciences and Infectious Diseases, Faculty of Veterinary Medicine and Animal Science, University of Life Sciences, 60-637 Poznan, Poland; emilian.malek@up.poznan.pl

**Keywords:** SARM, ostarine, muscle, C2C12 cells, L6 cells

## Abstract

Ostarine (also known as enobosarm or Gtx-024) belongs to the selective androgen receptor modulators (SARMs). It is a substance with an aryl-propionamide structure, classified as a non-steroidal compound that is not subjected to the typical steroid transformations of aromatization and reduction by α5 reductase. Despite ongoing research on ostarine, knowledge about it is still limited. Earlier studies indicated that ostarine may affect the metabolism of muscle tissue, but this mechanism has not been yet described. We aimed to investigate the effect of ostarine on the differentiation and metabolism of muscle. Using C2C12 and L6 cells, as well as muscles obtained from rats administered ostarine, we showed that ostarine stimulates C2C12 and L6 proliferation and cell viability and that this effect is mediated by androgen receptor (AR) and ERK1/2 kinase activation (*p* < 0.01). We also found that ostarine stimulates muscle cell differentiation by increasing myogenin, MyoD, and MyH expression in both types of cells (*p* < 0.01). Moreover, pharmacological blocking of AR inhibits the stimulatory effect of ostarine. We further demonstrated that 30 days of ostarine administration increases myogenin, MyoD, and MyH expression, as well as muscle mass, in rats (*p* < 0.01). Based on our research, we conclude that ostarine stimulates muscle tissue proliferation and differentiation via the androgen receptor.

## 1. Introduction

Loss of muscle mass (MM) is a problem connected to disease-related pathologies and accompanies old age. It can result in significant impairment of vital functions and significantly reduces quality of life [1]. Improved MM can be achieved in many ways, such as by maintaining a healthy lifestyle, physical exercise, and/or a proper diet containing, e.g., high-quality protein [2]. However, this is not always sufficient or possible due to the multitude of factors that influence these processes, such as diseases and hormonal disorders. One way to improve MM is to use steroid hormones, such as testosterone. The biological activity of testosterone (Ts) is regulated via the androgen receptor (AR), through which Ts affects muscle development and the maintenance and differentiation of new muscle fibers, among other processes [3]. The androgen receptor belongs to the group of nuclear receptors, and its natural ligands are testosterone and dihydrotestosterone. Disturbances in physiological concentrations of testosterone may be the result of primary hypogonadism or can occur in old age [4,5]. Moreover, the concentration of this hormone can change due to disease states and metabolic disorders, such as obesity and metabolic syndrome [6,7], and diseases, such as AIDS and cancer cachexia. Testosterone deficiency can cause many metabolic consequences, including metabolic disorder [8], obesity [9], and decreased bone mineral density [10] and can negatively affect social functioning by causing changes in behavior and mood [11]. That is why hormone replacement therapy (using testosterone) has been used in treatment and support for many years [12].

For almost 20 years, research has been carried out on developing effective therapeutic methods, during which adverse side effects typical of classic anabolic androgenic steroids have not been observed. Selective androgen receptor modulators (SARMs) are a group of compounds with various structures that have a strong anabolic effect but no adverse androgenic effects. However, research on the effectiveness and safety of such compounds is still ongoing [13]. Ostarine (also known as enobosarm or Gtx-024) is a SARM with an aryl-propionamide structure. It is a non-steroidal compound that is not subjected to transformations typical of steroids: aromatization and reduction by aromatase and 5-α reductase [13]. Ostarine has already undergone several phases of clinical trials for the treatment of sarcopenia, cachexia, and stress urinary incontinence [14]. At the time of our research, most clinical trials were either completed or were still in progress [15]. Despite continuing research, none of the SARMs, including ostarine, have yet been registered for medical purposes. However, the lack of registration is not an obstacle to the use of ostarine as a doping agent. Ostarine has been an item of interest to athletes, and although it is on the Prohibited List of the World Anti-Doping Agency, its use as a popular anabolic drug makes it necessary to better understand its effects (positive or negative) on the whole body, as well as individual cells and tissues.

Data in the literature show that ostarine improves bone healing [16] and modulates the functions of the uterus [14]. Our previous research also showed that ostarine was able to regulate the metabolism of isolated rat adipocytes [17]. As mentioned, ostarine is considered to be an agent that acts on the anabolic processes of muscles [18]. During muscle development and growth, inactive satellite cells activate to proliferate myoblasts; then, myoblasts multiply and begin the differentiation process to allow for the growth or regeneration of muscle tissue. Thus, new muscle fibers are formed by fusing mononuclear cells that form multinucleated myotubes, which then mature into muscle fibers with hundreds of cell nuclei [19]. Taking into account ostarine’s wide range of action, we became interested in its action in muscle tissue at the cellular level.

We decided to investigate the effect of ostarine on the proliferation, viability, and differentiation of C2C12 and L6 muscle cell lines, as well as the metabolism of skeletal muscles in vivo.

## 2. Results

### 2.1. Proliferation and Cell Viability

**Figure 1:**
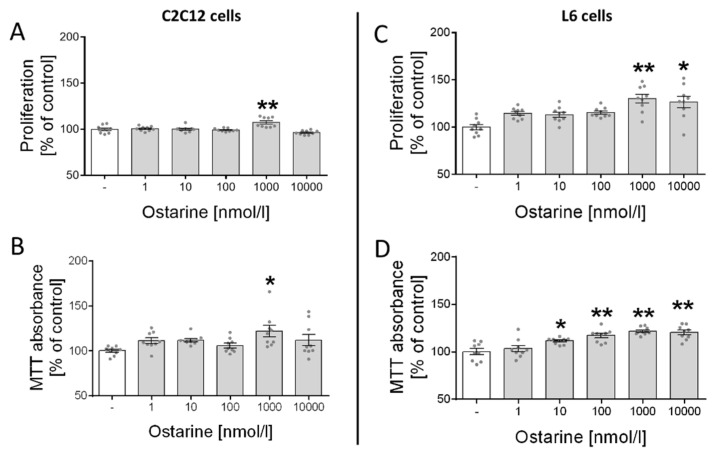
Effect of ostarine on (**A**,**C**) proliferation and (**B**,**D**) cell viability in C2C12 and L6 cells. Results are shown as percentage of control group (set to 100%), as mean ± standard error of mean (SEM) derived from *n* = 9 experiments (*n* = 3 per group with 3 repetitions of each). Statistically significant differences are represented as * *p* < 0.05 and ** *p* < 0.01 versus corresponding control using one-way ANOVA followed by Dunnett’s post hoc test.

First, we investigated the effect of ostarine on the proliferation and cell viability of C2C12 and L6 cells. We observed increased proliferation (*p* < 0.01; Figure 1A) and cell viability (*p* < 0.05; Figure 1B) in C2C12 cells after ostarine incubation at a dose of 1000 nM. We also found that ostarine had a stronger effect on L6 cells and significantly stimulated proliferation (1000 nM, *p* < 0.01; 10,000 nM, *p* < 0.05; Figure 1C) and cell viability (10 nM, *p* < 0.05; 100, 1000, and 10,000 nM, *p* < 0.01; Figure 1D) at lower doses.

**Figure 2:**
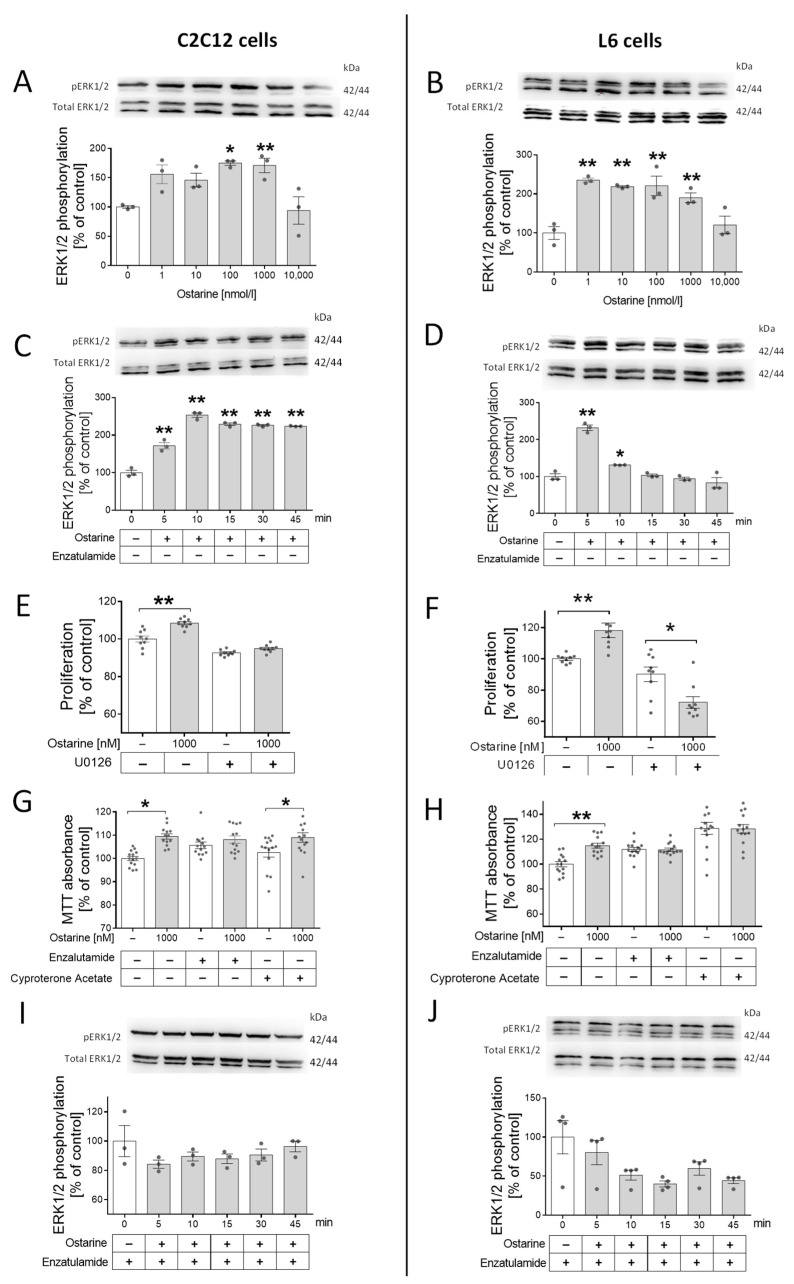
(**A**,**B**) Effect of different doses of ostarine on ERK1/2 phosphorylation in C2C12 and L6 cells. (**C**,**D**) Time activation of ERK1/2 phosphorylation by ostarine in C2C12 and L6 cells. (**E**,**F**) Impact of ostarine and ERK1/2 inhibition on proliferation of C2C12 and L6 cells. (**G**,**H**) Effect of ostarine and pharmacological AR blocking on viability of C2C12 and L6 cells. (**I**,**J**) Effect of ostarine on ERK1/2 phosphorylation in the presence of AR blocker enzalutamide in C2C12 and L6 cells. Statistically significant differences are represented as * *p* < 0.05 and ** *p* < 0.01 versus corresponding control using one-way ANOVA followed by Dunnett’s post hoc test (**A**–**D**,**I**,**J**) or unpaired Student’s *t*-test (two-tailed distribution) (**E**–**H**). Results are shown as mean ± standard error of mean (SEM) derived from *n* = 3 experiments (*n* = 1 per group with 3 repetitions of each) (**A**,**B**,**G**,**H**); *n* = 9 (*n* = 3 per group with 3 repetitions) (**C**,**D**); and *n* = 12–14 (*n* = 4–5 per group with 3 repetitions) (**E**,**F**).

The next step was to check whether this regulation was mediated by androgen receptor and ERK1/2 activation. We took this step because previous studies showed that the activation of ERK1/2 by steroid hormones can be mediated by AR without its activation. First, we noted that ERK1/2 was phosphorylated by different doses of ostarine in both cell lines (in C2C12, by 100 and 1000 nM, *p* < 0.01; in L6, by 1, 10, 100, and 1000 nM, *p* < 0.01; Figure 2A,B). Then, we investigated the time of ERK1/2 phosphorylation in C2C12 cells (*p* < 0.01; Figure 2C) and L6 cells (*p* < 0.01; Figure 2D). Then, using U0126, the pharmacological blocker of ERK1/2 kinase, we examined whether the proliferation stimulating effect was canceled after adding the inhibitor. Stimulation of the proliferation process by ostarine in cells treated with U0126 (C2C12 and L6, Figure 2E,F) was not observed.

Surprisingly, in L6 cells, the presence of ERK1/2 blocker in the incubation medium not only eliminated the stimulating effect of ostarine on the proliferation process but also significantly inhibited this process (*p* < 0.05; Figure 2F). We also noticed that adding AR blockers (enzalutamide and cyproterone acetate) abolished the effect of ostarine on cell viability. In C2C12 cells, a stimulating effect of ostarine on cell viability after adding enzalutamide (Figure 2G) was not observed; however, cyproterone acetate did not abolish the influence of this SARM (Figure 2G). In L6 cells, the stimulating effect of ostarine on cell viability was abolished after both AR blockers were added (Figure 2H). Based on previous experiments, we decided to confirm that stimulation of ERK1/2 phosphorylation is mediated by AR. After blocking AR using enzalutamide, we did not observe ERK1/2 phosphorylation in either experimental model (Figure 2I,J).

For this experiment, a serum-free medium (supplemented with 0.2% of BSA) was used in order to avoid the influence of other substances that are present in the serum, which was impossible in the case of investigation of the effect of ostarine on the differentiation process (initialized by addition of horse serum).

### 2.2. Differentiation Process in L6 Cells

**Figure 3:**
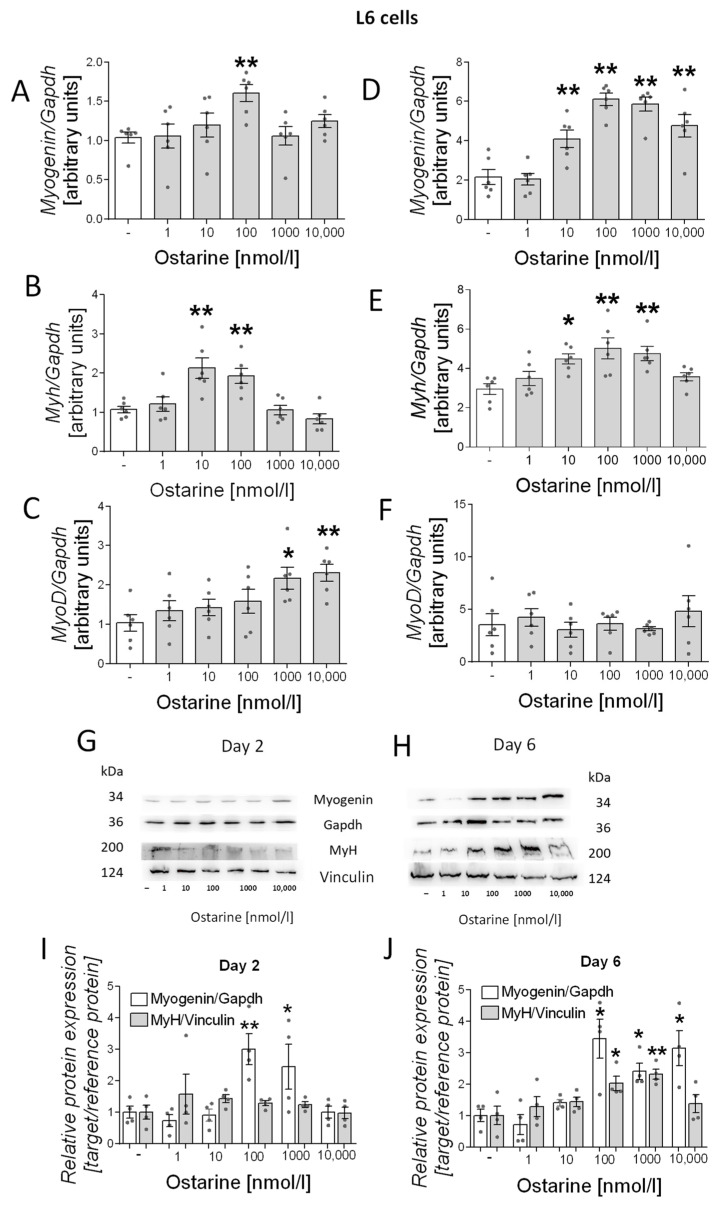
Effect of ostarine on L6 differentiation. mRNA expression of *myogenin*, *myh*, and *myoD* after (**A**–**C**) 2 days and (**D**–**F**) 6 days of differentiation initiation. (**G**,**H**) Effect of ostarine on myogenin and MyH protein level changes after 2 and 6 days of differentiation initiation and quantification of the amount of proteins in day 2 and day 6 (**I**,**J**). Statistically significant differences are represented as * *p* < 0.05 and ** *p* < 0.01 versus corresponding control using one-way ANOVA followed by Dunnett’s post hoc test.

Then, we investigated the effect of ostarine on the differentiation process in L6 cells. We observed increased mRNA expression of differentiation markers (*myogenin, myh*, and *myoD*) in early and late stages of differentiation (days 2 and 6 after initiation of the differentiation process). We observed increased mRNA expression of *myogenin* (100 nM, *p* < 0.01; Figure 3A), *myh* (10 and 100 nM, *p* < 0.01; Figure 3B), and *myoD* (1000 and 10,000 nM, *p* < 0.05 and *p* < 0.01, respectively; Figure 3C) after 2 days of differentiation and of *myogenin* (10, 100, 1000, and 10,000 nM, *p* < 0.01; Figure 3D) and *myh* (10 nM, *p* < 0.05; 100 and 1000 nM, *p* < 0.01; Figure 3E) after 6 days of differentiation. We also investigated the effect of ostarine on myogenin and MyH protein content. We observed increased protein content on day 6 of differentiation (Figure 3H), whereas after 2 days, the effect was less visible (Figure 3G). We also found that the addition of 100 and 1000 nM of ostarine stimulated myogenin protein expression after 2 days (Figure 3I; 100 nM, *p* < 0.01; 1000 nM, *p* < 0.05) of differentiation, whereas the addition 100, 1000, and 1000 nM of ostarine stimulated protein expression after 6 days (Figure 3J; *p* < 0.05). We did not observe an effect of ostarine on MyH protein level after 2 days (Figure 3I); however, after 6 days, we noted increased MyH in groups with the addition of 100 and 1000 nM (Figure 3J; 100 nM, *p* < 0.05; 1000 nM, *p* < 0.01).

### 2.3. Differentiation Process in C2C12 Cells

**Figure 4:**
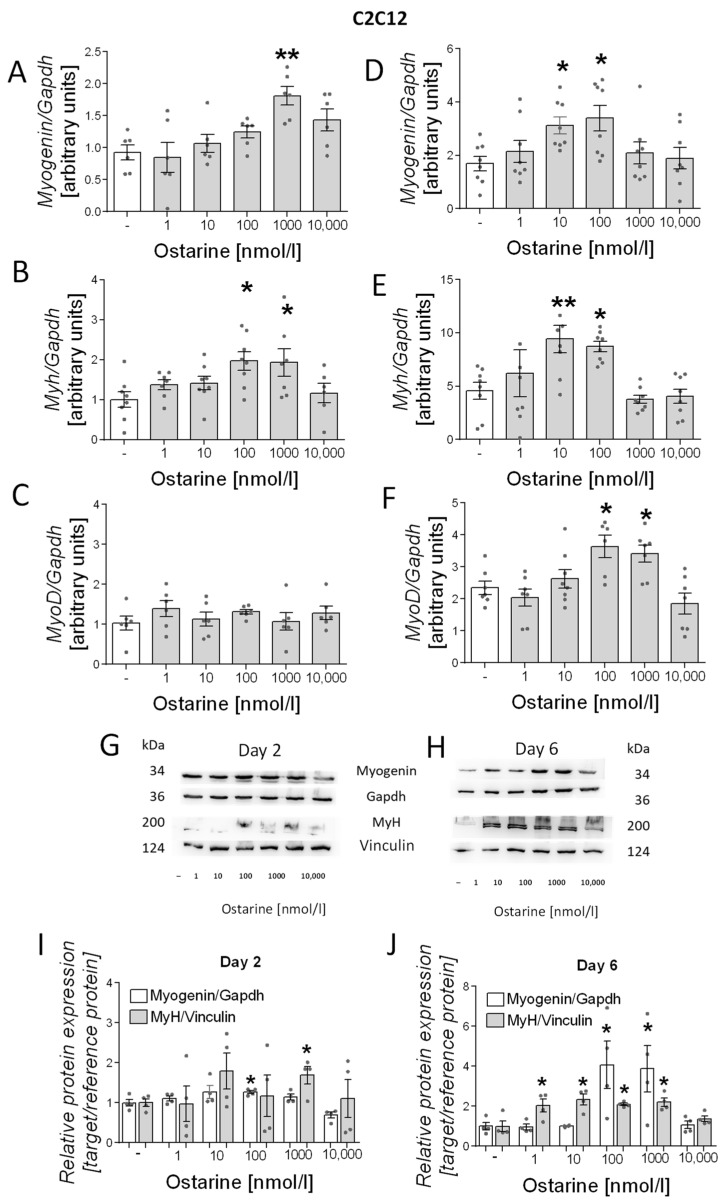
Effect of ostarine on C2C12 differentiation. mRNA expression of *myogenin*, *myh*, and *myoD* after (**A**–**C**) 2 days and (**D**–**F**) 6 days of differentiation initiation. (**G**,**H**) Effect ostarine on myogenin and MyH protein level changes after 2 and 6 days of differentiation initiation and quantification of the amount of proteins in day 2 and day 6 (**I**,**J**). Statistically significant differences are represented as * *p* < 0.05 and ** *p* < 0.01 versus corresponding control using one-way ANOVA followed by Dunnett’s post hoc test.

We also investigated the effect of ostarine on the differentiation process in C2C12 cells. As in L6 cells, we noted increased mRNA expression of differentiation markers (*myogenin, myh*, and *myoD*) in early and late stages of differentiation (days 2 and 6 after initiation). We observed increased mRNA expression of *myogenin* (1000 nM, *p* < 0.01; Figure 4A) and *myh* (100 and 1000 nM, *p* < 0.05; Figure 4B) after 2 days of differentiation and *myogenin* (10 and 100 nM, *p* < 0.05; Figure 4D), *myh* (10 and 100 nM, *p* < 0.01 and *p* < 0.05, respectively; Figure 4E), and *myoD* (100 and 1000 nM, *p* < 0.01 and *p* < 0.05, respectively; Figure 4F) after 6 days of differentiation. We observed increased protein content of myH on days 2 and 6 post-differentiation initiation; however, myogenin content was higher only on day 6 after ostarine treatment (Figure 4G,H). We also found that the addition of 100 nM of ostarine stimulated myogenin protein expression after 2 days (Figure 4I; 100 nM, *p* < 0.05) of differentiation, whereas the addition of 100 and 10,000 nM of ostarine stimulated protein expression after 6 days (Figure 4J; *p* < 0.05). Additionally, we observed a higher MyH protein level after 2 days in the group with the addition of 1000 nM of ostarine (Figure 4I; 1000 nM, *p* < 0.01), whereas after 6 days, we noted increased MyH after the addition of 1, 10, 100, and 1000 nM of ostarine (Figure 4J; *p* < 0.05).

### 2.4. Effect of Ostarine on Fusion Index in C2C12 and L6 Cells

**Figure 5:**
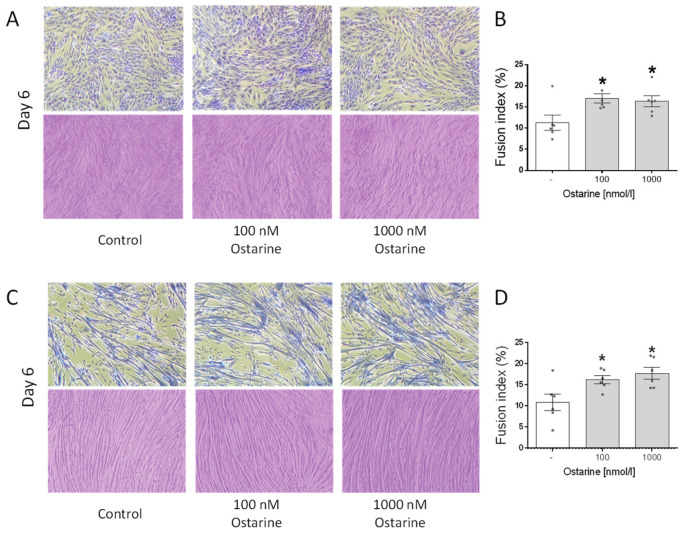
Representative light microscopic pictures of cells showing changes in cell morphology and Jenner–Giemsa staining after ostarine treatment after 6 days of differentiation in L6 (**A**) and C2C12 (**C**) cells. Fusion index changes after ostarine treatment in L6 (**B**) and C2C12 (**D**) cells. Statistically significant differences are represented as * *p* < 0.05 versus corresponding control using one-way ANOVA followed by Dunnett’s post hoc test.

Figure 5A shows illustrative pictures of L6 cells on day 6 of differentiation that were exposed to ostarine at 100 and 1000 nM doses, as well as Jenner–Giemsa staining, which was used to calculate the fusion index. We found that both of these doses increased the fusion index in L6 cells (*p* < 0.01; Figure 5B). Figure 5C shows illustrative pictures of C2C12 cells on day 6 of differentiation that were exposed to ostarine at 100 and 1000 nM doses, as well as Jenner–Giemsa staining, which was used to calculate the fusion index. We found that both doses increased the fusion index in L6 cells (*p* < 0.05; Figure 5D).

### 2.5. Effect of Ostarine Is Mediated by AR in C2C12 and L6 Cells

**Figure 6:**
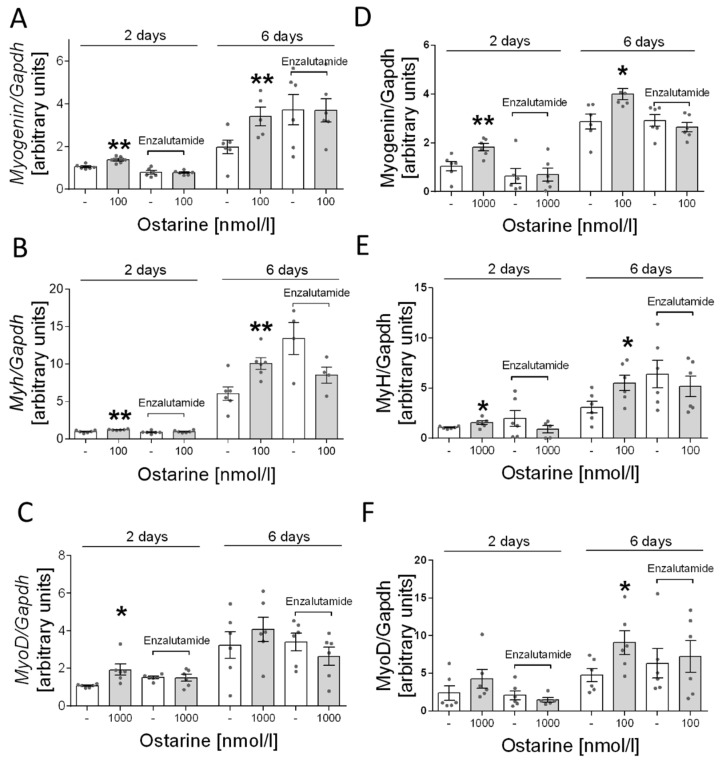
Effect of pharmacological blocking of AR and ostarine on *myogenin*, *myh*, and *myoD* mRNA after 2 and 6 days of differentiation initiation in (**A**–**C**) L6 cells and (**D**–**F**) C2C12 cells. Statistically significant differences are represented as * *p* < 0.05 and ** *p* < 0.01 versus corresponding control using unpaired Student’s *t*-test (two-tailed distribution) or Mann–Whitney U test.

Next, we determined whether the stimulation of ostarine-induced differentiation is mediated by AR receptor in L6 cells. Based on previous experiments, we selected the most effective dose of ostarine: 1000 nM. We found that adding enzalutamide inhibited the stimulating effect of ostarine on days 2 and 6 after initiation of differentiation (Figure 6A–C).

Furthermore, as in the case of L6 cells, we investigated whether the effect of ostarine is mediated only by the androgen receptor. In this case, also based on previous experiments, we selected the most effective doses of ostarine. Analogous to the results for L6 cells, we found that adding enzalutamide inhibited the stimulating effect of ostarine on days 2 and 6 after initiation of differentiation in C2C12 cells (Figure 6D–F).

### 2.6. Effect of Ostarine on Muscle Differentiation in Rats

**Figure 7:**
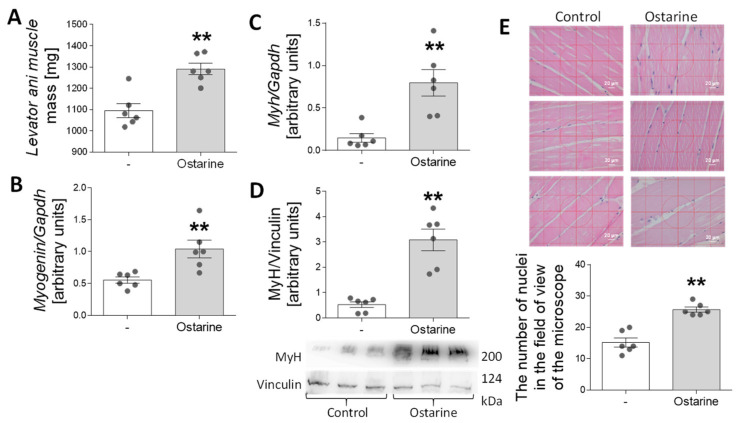
Effect of 30-day ostarine treatment on (**A**) levator ani muscle mass, (**B**,**C**) mRNA expression of *myogenin* and *myh*, and (**D**) protein level of MyH. (**E**) Changes in number of nuclei in microscopic muscle sections. Statistically significant differences are represented as ** *p* < 0.01 versus control group using unpaired Student’s *t*-test (two-tailed distribution) or Mann–Whitney U test (*n* = 6 per group).

In the last stage of the experiment, we investigated whether 30-day administration of ostarine to rats would increase the rate of muscle differentiation and affect muscle mass. We observed an increase in levator ani muscle mass after ostarine treatment (*p* < 0.01; Figure 7A). Moreover, we detected increased mRNA expression of *myogenin* (*p* < 0.01; Figure 7B) and *myh* (*p* < 0.01; Figure 7C), as well as MyH protein content (*p* < 0.01; Figure 7D). We also noted increased numbers of nuclei when rat muscle was stained after ostarine treatment (*p* < 0.01; Figure 7E). The levator ani was used as an indicator of the anabolic response of the skeletal muscle [20]. However, using it as a sole indicator may not be sufficient [21], so we decided to use the thigh muscle as a material for assessing changes in protein expression.

## 3. Discussion

In our study, we analyzed the effect of ostarine on cell differentiation in C2C12 and L6 cell lines as standard models for research into muscle physiology. We found that ostarine stimulated proliferation, cell viability, and differentiation of C2C12 and L6 cells. Moreover, we found that these effects were mediated by the androgen receptor.

Due to the fact that knowledge about the role of ostarine in cell proliferation and survival is limited, we decided to use both a method directly examining the proliferation process (i.e., BrdU) and MTT staining, by which we were able to indirectly determine that ostarine affects proliferation without also affecting cell death, e.g., by an excessively fast rate of multiplication and metabolism. As the results of the BrdU and MTT tests were complementary, we can conclude that ostarine modulates metabolism by influencing the proliferation rate without affecting cell death. The results stand in opposition to those obtained using breast cancer cells, where ostarine inhibits proliferation of these cells. This could be explained, however, by the fact that androgens inhibit cellular proliferation in breast tissue in different types of cells (normal vs. cancer cell) [20,22].

Ostarine is a compound that selectively activates the androgen receptor. Although it has been studied in clinical trials, it has still not received Food and Drug Administration (FDA) approval [23]. This SARM has gained popularity due to its pharmacokinetic properties; as a non-steroidal agent, it does not undergo classical metabolic pathways characteristic of steroid compounds, e.g., testosterone and its derivatives [13]. The classical mechanism of action of androgens is based on signaling through the androgen receptor, which stimulates the androgen response element (ARE) in the genome [6]. However, it is known that androgens also act through non-genomic action by activation of MAPK kinase pathways [24,25]. Ostarine, unlike other naturally occurring androgens, such as testosterone, is a compound that selectively activates the AR and belongs to the group of compounds whose development makes it possible to avoid adverse effects on receptors located in androgenic tissues, e.g., prostate.

It turns out that ostarine can activate non-genomic pathways [18]. Our study shows that the effects on the proliferation and survival of cell lines are related to AR activation, as its blockade by the second-generation inhibitor enzalutamide is abolished. Proliferation and cell viability may be regulated by ERK kinases [26]. Moreover, AR and ERK signaling are interrelated. It is also known that a natural ligand of the AR, such as testosterone or dihydrotestosterone, can act on this kinase phosphorylation in two ways: via the AR receptor or the G-protein-coupled receptor [27,28]. That is why we decided to use an AR inhibitor to determine whether the AR also mediates ERK kinase activation in the tested model. Using an AR inhibitor, we observed blocked proliferation, although this effect was only seen with enzalutamide. Enzalutamide is a second-generation antiandrogen compound with a stronger AR blocking effect, as it inhibits not only receptor binding but also AR nuclear translocation and AR interaction with coactivators [29]. In our study, enzalutamide additionally blocked the phosphorylation of ERK kinases, which confirms the relationship between ERK and AR. The pathway of action of ostarine in this case is directed only in the AR. This confirmed the selectivity of ostarine for its action on cells. The use of U0126 inhibitor also adversely affects survival and proliferation. This blocking effect was observed in L6 cells in the proliferation process. L6 cells differ from C2C12 cells with respect to AR activation, as proven by Fu et al. [30], and our research confirms that activation in L6 is more strongly associated with kinase activation. This suggests that in the case of muscle myotubes, both AR and kinase blocking can adversely affect the physiological features of analyzed cells.

The second aspect we analyzed was the effect of ostarine on myotube differentiation. Fully developed skeletal muscle is made of stable, multinuclear tissue that has the ability to regenerate. Repair processes are the basis of fiber regeneration but can also contribute to muscle growth in response to physical exercise. The regeneration of muscle fibers and the formation of new fibers are complex processes that involve a number of molecular processes. It is well known that testosterone is responsible for the development of new muscle fibers, and in this case, a key signaling element in the process is signaling through the androgen receptor. This was proven in a study showing that AR expression increases during differentiation. In addition, a high dose of testosterone was shown to cause a significant increase in AR expression [31]. However, it has also been shown that androgens may have non-genomic effects in skeletal muscle [32,33].

There is a limitation in comparing selective modulators. Selective modulators comprise a whole range of compounds, and slight modifications affect their activity and specificity [34]. However, according to the available literature, it can be seen that the steroidal SARM YK11 stimulates C2C12 cell differentiation. A similar effect was seen with another synthetic SARM [35]. The available data on cell lines in the literature are insufficient to be able to compare dose data. In our study, we showed that the process of early-stage myoblast proliferation and differentiation into mature myotubes is dose-dependent. Kitakaze et al. obtained a similar effect in lactoferrin studies, where the differentiation of myotubes was not dependent on the highest dose, whereas proliferation was dose-dependent [36]. In studies with the phytoestrogen genistein, it was shown that this compound also promotes proliferation and differentiation at various doses [37]. The signaling pathways for the two processes are different; thus, there may be divergent results, but they require further research. Our research confirmed the anabolic effects of ostarine in vivo. In contrast to studies on the cell line model, the increased muscle protein expression could also result from the activation of satellite cells, the proliferation and differentiation of which result in more muscle fibers. It is worth noting that the study was based on a low dose of ostarine and lasted 30 days. Ostarine has also been studied for the metabolism of bone tissue and muscle mass in ovariectomized female rats. Contrary to our study results, no hypertrophy was observed in the muscles of female rats, which may be due to the increased presence of AR in males compared to females [38].

An important limitation of our study is that we did not identify the intracellular pathways that confer the effects of ostarine on muscle cell differentiation; furthermore, we used only pharmacological blocking of the androgen receptor to investigating its role in ostarine’s effect on L6 and C2C12 cells. An important limitation of our study is the lack of in vivo evidence using both rat and mouse models, as well as the use of only one gender, which may be important, especially in the case of research concerning androgens. However, as this is the first study of this type, we decided to use males, as the sex with higher susceptibility to the effects of androgens. We are also aware that a detailed understanding of the role of ostarine in the metabolism of muscle and other tissues requires further research, especially if the results are to be used in clinical treatment.

In summary, we found that ostarine stimulates proliferation, cell viability, and differentiation of muscle cells, and these results clearly indicate that ostarine can be considered as a potential modulator of these processes in muscle tissue.

## 4. Materials and Methods

### 4.1. Animals

Wistar rats were purchased from Mossakowski Medical Research Centre, Polish Academy of Science (Warsaw, Poland). All procedures performed in this study involving animals were in accordance with ethical standards. The study was also approved by the Local Ethical Commission for Investigations on Animals (permission no. 39/2019).

### 4.2. Ostarine Injections

Male Wistar rats (*n* = 36) were acclimatized for 1 week, and then ostarine was administered by subcutaneous injection at a dose of 0.4 mg/kg body weight (bw) determined from the literature [39]. Ostarine was dissolved in ethanol PEG300 as vehicle (10:90 (*v*:*v*)). Rats were injected with ostarine every day for 30 consecutive days. Animals in the control group received equal amounts of vehicle.

### 4.3. Chemicals

Media and supplements were from Corning (Tewksbury, MA, USA). Ostarine (purity 99.36%) was purchased from Selleck Chemicals (No. S1174). Antibodies were as follows: anti-myogenin, anti-myh, anti-myoD, anti-vinculin, anti-phospho-ERK1/2, and anti-ERK1/2 were from Santa Cruz Biotechnology (Dallas, TX, USA). Anti-GAPDH and all secondary antibodies for Western blot were from Sigma-Aldrich (Taufkirchen, Germany). The AR blockers used were as follows: enzalutamide was from Selleck Chemicals (No. S1250), and cyproterone acetate was from Santa Cruz Biotechnology (sc-204703). Unless otherwise stated, all other reagents were from Sigma-Aldrich.

### 4.4. C2C12 and L6 Cell Culturing

C2C12 and L6 cell lines were purchased from the European Collection of Authenticated Cell Cultures (ECACC). Both cell lines were grown under the same conditions. Undifferentiated cells were cultured according to the manufacturer’s instructions. Cells were cultured in a standard culture medium (Dulbecco’s Modified Eagle Medium (DMEM) containing 4.5 g/L glucose and L-glutamine supplemented with 10% fetal bovine serum (FBS) and 1% penicillin–streptomycin) at 37 °C in a humidified 5% CO_2_ incubator.

The experimental medium was DMEM with the addition of 0.2% bovine serum albumin (BSA) instead of FBS.

### 4.5. C2C12 and L6 Cell Differentiation

Cells seeded on 6- or 12-well plates were cultured in DMEM with 10% FBS to 80–90% confluence; then, to initiate myogenic differentiation, the medium was changed to DMEM supplemented with 2% horse serum (HS) and different concentrations of ostarine [40,41]. Cells were collected 2 days (early stage) and 6 days (late stage) after initiation of the differentiation process.

### 4.6. Proliferation and Cell Viability

Cells were seeded onto a 96-well plate and cultured in a standard culture medium for 24 h. Then, cells were washed in PBS, and the medium was changed to experimental (DMEM with 0.2% BSA), supplemented with different concentrations of ostarine, and cultured for the next 24 h. Some cells were grown in standard medium as a positive control.

After 24 h, to investigate viability, cells were treated with MTT solution ((3-(4,5-dimethylthiazol-2-yl)-2,5-diphenyltetrazolium bromide) for 1 h. Then, the medium was removed, and 100 μL of DMSO was added to the cells to dissolve the formazan crystals. The optical density was measured using a Synergy 2 microplate reader (BioTek Instruments, Winooski, VT, USA).

Cell Proliferation ELISA BrdU colorimetric kit (Roche Diagnostics, Penzberg, Germany) was used to test proliferation by evaluating the incorporation of bromodeoxyuridine (BrdU) into new DNA strands of proliferating cells. After 21 h incubation with the examined concentrations of ostarine, BrdU solution (10 μM) was added for 3 h. The assay was performed according to the manufacturer’s instructions. The optical density of these samples was also measured using a Synergy 2 microplate reader (BioTek Instruments, Winooski, VT, USA).

### 4.7. RNA and Real-Time qPCR

Total RNA from cells was isolated using Extrazol reagent according to the manufacturer’s instructions (DNA Gdansk, Poland). cDNA was generated using 1 µg of total RNA with a high-capacity cDNA reverse transcription kit (Life Technologies, Grand Island, NY, USA). Real-time qPCR was performed using a Quant Studio 12K Flex™ system. GAPDH was used as the reference gene. (Primer sequence are listed in Table 1).

### 4.8. Western Blot Analysis

Western blot analysis was performed as previously described [42].

### 4.9. Tissue Staining

The quadriceps muscles were collected in 10% paraformaldehyde, and sections were prepared. Paraffin sections were collected on microscope slides (Menzel-Glaser, SuperFrost Plus, Thermo Scientific, Budapest, Hungary) and then stained using hematoxylin and eosin stain.

### 4.10. Jenner–Giemsa Staining

Cells were seeded on 6-well plates. When cells reached 90% confluence, the growth medium was replaced with a differentiation medium: DMEM containing 2% horse serum supplemented with ostarine in 100 and 1000 nM concentrations. After the 6th day of differentiation, the cells were fixed by methanol. After removal of methanol, cells were incubated with Jenner staining solution prepared in a 1:3 ratio from stock solution and buffered sodium phosphate. The stock solution was 1 g of Jenner’s dye in 400 mL of methanol. After 10 min of incubation in the staining solution, the cells were rinsed with distilled water, and Giemsa staining solution was added. The Giemsa solution was diluted 20× with buffered sodium phosphate. After 10 min incubation, cells were washed twice with distilled water. The fusion index was calculated by staining the fixed cells and determining the number of nuclei in a myotubule compared to the total number of nuclei in the sample using an inverted Delta Optical IB-100 light microscope.

## Figures and Tables

**Table 1:** Primer sequences and product sizes.

Target	Forward Primer (5′ > 3′)	Reverse Primer (5′ > 3′)	Product (bp)
*Myogenin (Rattus)*	CTACAGGCCTTGCTCAGCTC	TGGGAGTTGCATTCACTGG	101
*MyoD (Rattus)*	ACTACAGCGGCGACTCAGAC	ACTGTAGTAGGCGGCGTCGT	122
*Myh3 (Rattus)*	GCAGAGACCATCAAGCACCT	GTGCAGCTGGGTGTCCTT	60
*GAPDH (Rattus)*	CTGCACCACCAACTGCTTAG	TGATGGCATGGACTGTGG	92
*Myogenin (Mus)*	CGGTGGAGGATATGTCTGTTG	GGTGTTAGCCTTATGTGAATGG	215
*Myh3 (Mus)*	ATGAGCGGCGTGTTAAGGA	ATTGACTTGCGATTCTGCGATA	227
*MyoD (Mus)*	AGCACTACAGTGGCGACTCA	GGCCGCTGTAATCCATCAT	75
*GAPDH (Mus)*	ATGGTGAAGGTCGGTGTGA	AATCTCCACTTTGCCACTGC	84

## Data Availability

The data presented in this study are available on reasonable request from the corresponding author.
